# Cavitation Induced by Janus-Like Mesoporous Silicon Nanoparticles Enhances Ultrasound Hyperthermia

**DOI:** 10.3389/fchem.2019.00393

**Published:** 2019-06-05

**Authors:** Andrey Sviridov, Konstantin Tamarov, Ivan Fesenko, Wujun Xu, Valery Andreev, Victor Timoshenko, Vesa-Pekka Lehto

**Affiliations:** ^1^Faculty of Physics, M. V. Lomonosov Moscow State University, Moscow, Russia; ^2^Department of Applied Physics, University of Eastern Finland, Kuopio, Finland; ^3^Institute of Engineering Physics for Biomedicine, National Research Nuclear University MEPhI, Moscow, Russia; ^4^Lebedev Physical Institute of the Russian Academy of Sciences, Moscow, Russia

**Keywords:** porous silicon, nanoparticles, therapeutic ultrasound, hyperthermia, heating, selective modification, hydrophobic, hydrophilic

## Abstract

The presence of nanoparticles lowers the levels of ultrasound (US) intensity needed to achieve the therapeutic effect and improves the contrast between healthy and pathological tissues. Here, we evaluate the role of two main mechanisms that contribute to the US-induced heating of aqueous suspensions of biodegradable nanoparticles (NPs) of mesoporous silicon prepared by electrochemical etching of heavily boron-doped crystalline silicon wafers in a hydrofluoric acid solution. The first mechanism is associated with an increase of the attenuation of US in the presence of NPs due to additional scattering and viscous dissipation, which was numerically simulated and compared to the experimental data. The second mechanism is caused by acoustic cavitation leading to intense bubble collapse and energy release in the vicinity of NPs. This effect is found to be pronounced for as-called Janus NPs produced via a nano-stopper technique, which allow us to prepare mesoporous NPs with hydrophobic inner pore walls and hydrophilic external surface. Such Janus-like NPs trap air inside the pores when dispersed in water. The precise measurement of the heating dynamics *in situ* enabled us to detect the excessive heat production by Janus-like NPs over their completely hydrophilic counterparts. The excessive heat is attributed to the high intensity cavitation in the suspension of Janus-like NPs. The present work elicits the potential of specifically designed Janus-like mesoporous silicon NPs in the field of nanotheranostics based on ultrasound radiation.

## Introduction

Hyperthermia is considered to be one of the most popular and well-studied treatment modalities in cancer therapy (Wust et al., 2002). This non-ionizing treatment is based on the temperature rise in the tumor, that makes cancer cells more susceptible to radio- and chemotherapy due to the heat-induced chemosensitization (Issels, 2008) and tumor tissue oxygenation (Song et al., 2001). Furthermore, some specific proteins provide healthy and malignant cells with different hyperthermal sensitivity leading to the selective destruction of the tumor (Setroikromo et al., 2007).

There are several methods to produce hyperthermia, but the general aim is to generate hyperthermia locally with external electromagnetic radiation (Tamarov et al., 2014) or ultrasound (US) (Diederich and Hynynen, 1999) sources. US, being widely used in modern medicine (Hill et al., 2005), is one of the most promising and effective external sources for the local hyperthermia. This is due to its numerous advantages over laser or electromagnetic wave sources: low invasiveness, precise focusing, and excellent penetration depth. These features are crucial for the local thermo-ablation of both benign and malignant tumors deep inside the body without causing serious harm to the skin or the adjacent healthy tissues (Bessonova et al., 2009; Miller et al., 2012).

Currently available US-mediated treatment techniques employ high-intensity focused ultrasound (HIFU) sources, which enable a relatively high degree of accuracy and agility (Frazier et al., 2006; Tempany et al., 2011). However, the utilization of HIFU has several drawbacks. In the technique, the precise and expensive focusing equipment is required to achieve the absorption contrast between the normal tissues and the lesion sites (Haar and Coussios, 2007). Due to the small focal region of HIFU, the volume of the tissue to be ablated is limited leading to long exposure times, which can cause undesired thermal injuries and DNA mutations in the surrounding healthy tissues due to the US waves reflected from interfaces (Chatterjee et al., 2011).

Special micro- and nanoagents, sometimes referred as sonosensitizers (Deepagan et al., 2016), were proposed to overcome the limitations of HIFU ablation. Sonosensitizers can significantly enhance the therapeutic efficiency by reducing the US intensity and duration necessary for the therapeutic effect (Sviridov et al., 2015; Kosheleva et al., 2016). The recent advances in nanobiotechnology and nanomedicine have directed the researchers to create various types of sonosensitizers. For example, solid gold (Kosheleva et al., 2016), magnetic (Józefczak et al., 2016), and silicon (Sviridov et al., 2015) NPs, as well as highly heat-conducting graphene oxide nanosheets (Darabdhara et al., 2015), are good mediators for the US-induced hyperthermia because of significant energy release via the US absorption. Mesoporous silicon-based NPs enhance the cavitation effect of US (Kharin et al., 2015; Sviridov et al., 2015), which leads to the additional heating of the surroundings and initiation of sonochemical reactions and sonoluminescence, i.e., light generation (Qian et al., 2016). TiO_2_, polyhydroxy fullerene, alumina, and platinum NPs can respond to US by generating reactive oxygen species for the sonodynamic therapy (Serpe et al., 2012; Canavese et al., 2018; Pan et al., 2018). Furthermore, different nanocomposites and nanocontainers can be used to enhance synergistic effects of US treatment modalities as well (Qian et al., 2017).

Biomedical applications of solid sonosensitizers are usually limited due to their cytotoxicity and low biodegradability (Yildirimer et al., 2011). In this regard, biocompatible and biodegradable porous silicon (PSi) NPs seem to be very promising material for various medical applications (Sviridov et al., 2017). We have previously demonstrated that the presence of NPs led to the temperature difference between the aqueous PSi NP suspensions and distilled water under US irradiation (Sviridov et al., 2013). There, the high heating efficiency was achieved in the acoustic resonator, which increased the US wave amplitude by several orders of magnitude. Unfortunately, the geometry of resonator is not applicable *in vivo*.

In our recent study, Janus-like PSi NPs were designed to be specifically employed with ultrasound. In these NPs, the pore walls were hydrophobic and the external surfaces of NPs were hydrophilic giving sustainability in aqueous solution while maintaining air inside the pores (Tamarov et al., 2017). The present work compares the enhanced heating effects produced by fully oxidized, hydrophilic PSi NPs (O-PSi NPs), and Janus-like PSi NPs (J-PSi NPs) in the field of the US traveling wave, and it explains the phenomenology behind the exceptional cavitation and heating capability of these NPs. The pronounced temperature rise in the area of the NP localization can be applied to the hyperthermia treatment of tumors, or for the purposes of drug release from PSi NPs coated with thermo-sensitive polymers (Tamarov et al., 2016).

## Materials and Methods

### Materials

Si wafers (diameter 20 cm, p^+^ (100), 0.01–0.02 Ω·cm, Okmetic Inc.), ethanol (EtOH, absolute, Altia Oyj), ammonium hydroxide (NH_4_OH, 28%, VWR), hydrogen peroxide (H_2_O_2_, >30% w/v, Fisher Scientific), n-hexane (≥99%, Merck), toluene (anhydrous, 99.8%, Alfa Aesar), hydrofluoric acid (38%, Merck) were used as received.

### Preparation of PSi NPs

**Figure 1:**
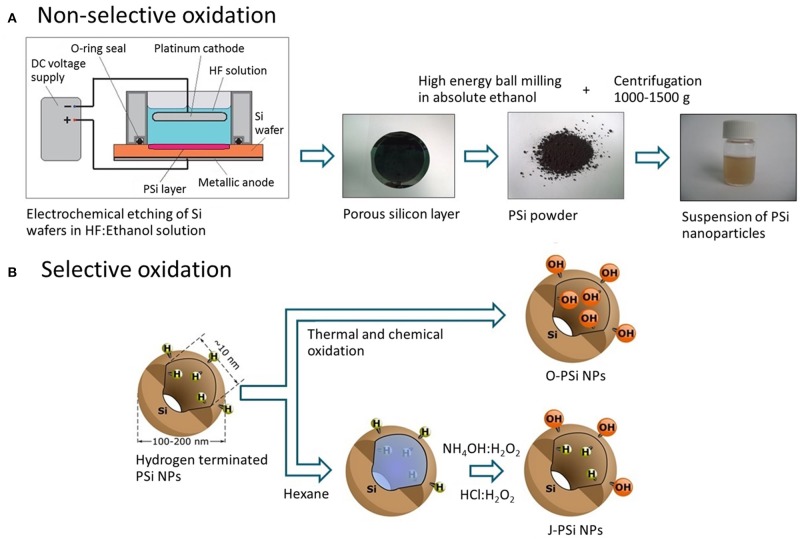
**(A)** Fabrication route of PSi NPs: Electrochemical etching of the silicon wafer, grinding of the free-standing PSi film, ball milling of the PSi powder in ethanol, and centrifugation to obtain the nanoparticle suspension. **(B)** Techniques of the PSi NP surface modification: Thermal oxidization (O-PSi NPs) and selective modification (J-PSi NPs) using hexane as a nano-stopper.

First, free-standing PSi (pore size ~10 nm) films were prepared by anodizing p^+^-type silicon wafers (100) with the resistivity of 0.01–0.02 Ω·cm in a HF (38%)-ethanol mixture (Bimbo et al., 2010). After drying at 65°C for 1 h, the obtained films were ground in a mortar and then ball milled in ethanol to produce PSi NPs. The overall sequence of preparation steps is depicted in Figure 1A.

In order to prepare J-PSi NPs, hexane was used as a nano-stopper (Xu et al., 2014; Tamarov et al., 2017) to protect hydrogen terminated (hydrophobic) pore walls that were the result of etching. Second, the unprotected outer surface was selectively oxidized in NH_4_OH:H_2_O_2_ (30%):H_2_O 1:1:6 at RT for 15 min and in HCl:H_2_O_2_ (30%):H_2_O 1:1:6 for 15 min, subsequently. This is possible since hexane is a non-polar solvent and does not mix with aqueous solutions. Next, PSi NPs were washed in water and stored in absolute ethanol. As a reference sample, fully hydrophilic nanoparticles were prepared through non-selective oxidization of PSi nanoparticles as described previously (Näkki et al., 2015) and are denoted as O-PSi NPs. These ways of surface modification are shown in Figure 1B.

### Characterization of PSi NPs

The surface composition after each step of the surface modification was verified with FTIR transmittance (Thermo Nicolet Nexus 8700) measurements of the KBr tablets containing the PSi NPs. The morphology of PSi NPs was imaged with transmission electron microscopy (TEM) in a JEOL JEM-2100F microscope. Dynamic light scattering (DLS, Malvern Instruments Zetasizer Nano ZS) was used to measure the hydrodynamic diameters of the NP samples in deionized water. Diluted suspensions (<0.2 mg/ml) were equilibrated at 25°C for 5 min prior to the measurements.

### Measurements of Heating

**Figure 2:**
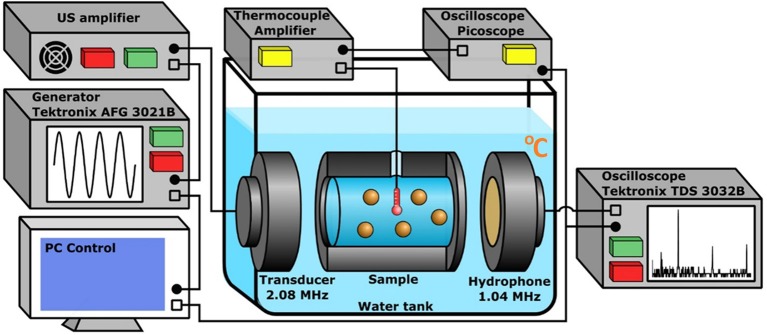
The experimental setup used for the heating measurement and cavitation detection in aqueous suspensions of PSi NPs. The cuvette, US transducer, and hydrophone are immersed into the water tank. The amplified sinusoidal signal from the generator is irradiated by the transducer, the oscilloscope collects data from the hydrophone and performs FFT. The E-type thermocouple measures the heating. The PC reads data from the oscilloscopes and controls the generator.

A specific setup was developed for the measurement of heating in the suspensions of PSi NPs. The core part of the setup consisted of an aluminum cylindrical sample chamber (cuvette) with two US transparent windows located between a flat transducer and a hydrophone immersed into a water tank (Figure 2). The volume of the cuvette was ~15 ml. The transducer with a diameter of 10 mm emitted a low-divergence US beam of frequency 2,080 kHz, which heated the samples. The diameter of windows was 25 mm and coincided with the internal diameter of the cuvette of 30 mm length. The distance between the transducer and cuvette was 10 mm, while the distance from the cuvette to the hydrophone was 50 mm. The transducer was connected to a custom-made US amplifier (output power of 75 W at 50 Ohms load in the frequency band of 1–5 MHz), which amplified the sinusoidal signal from a generator (Tektronix AFG 3021B). The matching of the output impedance of the amplifier with the piezotransducer was provided by trimming inductance. Simultaneously with temperature, the cavitation intensity was measured by detecting the subharmonic magnitude of US wave passing through the cuvette. For this purpose, an oscilloscope (Tektronix TDS 3032B) recorded a signal from the hydrophone within 0.1 ms, averaged it over 32 successive realizations and calculated the spectrum of the averaged signal. Hamming function was chosen as a time interval window. The generator and oscilloscope were connected to a PC with a custom NI™ LabVIEW 2013 (National Instruments Corp., Austin, TX) program to control the experiment. The obtained spectra were recorded with a repetition rate of 3 Hz.

The temperature was measured with a highly sensitive chromel-constantan E-type thermocouple (TC, OMEGA Engineering) encased in the cylindrical sheath with a diameter of 0.25 mm. The TC was inserted into the cuvette center. The TC signal was amplified by a self-made amplifier powered by a battery, which significantly lowered the level of electromagnetic noise. The amplified TC signal was registered by an oscilloscope (PicoScope 5204) and then loaded into the PC memory for storage and subsequent processing. The TC was calibrated in advance for the temperature range of 20–40°C using a mercury thermometer. The used TC model (thermocouple in a thin metal tube with earthing, EMQSS-010G-12) has a minimum response time of 0.2 s (Figure S1), which makes it possible to measure not only slow temperature trends, but also its rapid fluctuations. Special control experiments were carried out to ensure that the metal tube with the TC itself did not enhance cavitation in the investigated ranges of US power and concentration of PSi NPs. The collapse of cavitation bubbles on the surface of PSi NPs creates their overheating, which in turn leads to local heating of the medium. Such temperature fluctuations are rather random, since their magnitude is defined by the number of cavitation bubbles near the TC. The duration of local fluctuations is small, so a quick-response thermocouple is required to register them.

### Cavitation Measurements

Cavitation is known to be an important factor which influences the process of heating in aqueous suspensions and biological media. The threshold and intensity of acoustic cavitation can be determined by the appearance of subharmonic component and its magnitude in the spectrum of the signal recorded by the hydrophone (Didenkulov et al., 2011). The setup described above (see Figure 2) enabled the initiation and detection of cavitation in the suspensions of PSi NPs. In order to specifically amplify the subharmonic signal, the resonance frequency of the hydrophone was half (1.04 MHz) of the resonance frequency of the transducer (2.08 MHz). The cavitation threshold was measured by detecting the pressure amplitude, which corresponded to an abrupt growth of subharmonic magnitude extracted from the spectrum along with the fundamental and high-order harmonics (Figure S3A). The voltage on the transducer was increased stepwise to provide a change of acoustic pressure in the range of 0.3–0.5 MPa (Figure S4). Such pressure amplitudes were beyond the cavitation thresholds for the suspensions of Si NPs and water (Tamarov et al., 2017), because the level of subharmonic (in dB) remained much higher than the noise level throughout the period of exposure (blue dashed line in Figure S3B). At each pressure, the harmonics were measured for 200 s and their average values for this period were calculated to obtain the mean magnitudes (red dashed line in Figure S3B). Relative errors for each pressure were calculated as mean square deviations of normal distributions of the subharmonic magnitude smoothed by 10 points (Figure S3C).

## Numerical Calculation of Temperature Growth

The absorption of US energy in aqueous suspensions is associated with the relative motion of solid nanoparticles under the US wave field. In a viscous medium, the Stokes force is exerted on a particle leading to inconvertible heat loses, which depend on the ratio of the particle and medium densities, particle size and medium viscosity. The temperature ***T*** of the medium in which the US wave of intensity ***I*** propagates can be calculated using the heat transfer equation:

(1) $$\begin{array}{r} \frac{\partial T}{\partial t}\;=\chi \nabla^{2}T\;+\;\frac{2\alpha I}{\rho_{0}c_{p}}, \end{array}$$

where ***t*** is the time variable, **∇^2^** is Laplacian operator, **χ = κ*/*ρ_0_*c***_***p***_ is the thermal diffusivity, **κ** is the thermal conductivity, **α** is the US absorption coefficient in the medium, ***I*** is the US intensity, **ρ_0_** and ***c***_***p***_ are the medium density and the specific heat capacity, respectively.

To calculate the heating for a given set of parameters using Equation (1), one needs first to define the absorption coefficient **α**. The work of R. J. Urick is considered to be a classic work which estimated the attenuation coefficient of nanoparticle suspensions (Urick, 1948). According to this work, the attenuation coefficient **α_*a*_** of US wave of frequency **ω** in a medium with spherical particles of radius ***a*** can be written as a sum of two terms. The first term describes the process of wave scattering and is negligibly small for nano-sized particles, while the second one is associated directly with the absorption:

(2) $$\begin{array}{r} \alpha_{a}\;=\;\alpha_{w}\;+\;\frac{C}{2}\cdot \left[\frac{1}{6}k^{4}a^{3}\;+\;k{\left(\theta -1\right)}^{2}\frac{s}{s^{2}\;+\;{\left(\theta \;+\;\tau \right)}^{2}}\right], \end{array}$$

where the following notations are used:

$$\begin{array}{l} s\;=\;\frac{9}{4\beta a}\left(1\;+\;\frac{1}{\beta a}\right),\;\tau \;=\;\frac{1}{2}\;+\;\frac{9}{4\beta a},\;\theta \;=\;\frac{\rho_{1}}{\rho_{0}},\;\beta \;=\;{\left(\frac{\omega }{2\mu }\right)}^{\frac{1}{2}},\\ \;C=\frac{4}{3}\pi a^{3}n=\frac{\nu }{\rho_{1}}, \end{array}$$

***k* = *2*π*f/c*** is the wavenumber, ***c*** is the speed of sound in the medium, **μ** is the kinematic viscosity coefficient, **ρ_0_** is the medium density, **ρ_1_** is the nanoparticle density, ***C*** is the volume concentration of nanoparticles. The absorption coefficient of water **α_*w*_** for the MHz range of US frequencies ***f*** can be estimated using the following formula:

(3) $$\begin{array}{r} \alpha_{w}\;=\;\alpha_{1w}\;\cdot \;f^{2}, \end{array}$$

where **α_1*w*_** is the absorption coefficient of water for the frequency of 1 MHz, the frequency ***f*** is expressed in MHz.

The heating of an aqueous suspension exposed to US radiation during time ***t***_**1**_ can be calculated using a 1D heat transfer equation due to an axial symmetry of the experimental setup (see Figure 2; Figure S2):

(4) $$\begin{array}{r} \begin{array}{c} \frac{\partial T}{\partial t}\;=\chi \;\frac{\partial^{2}T}{\partial r^{2}}+\frac{2\alpha I}{\rho_{0}c_{p}},\;{0<r\;\leq R}_{\mathrm{beam}},\\ \;\frac{\partial T}{\partial t}=\chi \;\frac{\partial^{2}T}{\partial r^{2}},\;R_{\mathrm{beam}}<r<R_{\mathrm{cuv}}, \end{array} \end{array}$$

where ***r*** is the radial coordinate, ***R***_***beam***_ is the US beam radius, ***R***_***cuv***_ is the radius of the cuvette filled with the suspension. The physical constants and model parameters are enumerated in Table S1. The initial and first-type (Dirichlet) boundary conditions are:

(5a) $$\begin{array}{r} t=0:\;T=T_{0},\;0\;\leq r\;\leq R_{\mathrm{cuv}}; \end{array}$$

(5b) $$\begin{array}{r} r=0:-\chi \rho_{0}c_{p}\frac{\partial T}{\partial r}=0,\;0<t\leq t_{1}; \end{array}$$

(5c) $$\begin{array}{r} r=R_{\mathrm{cuv}}:\;T=T_{0},\;0<t\leq t_{1}, \end{array}$$

where ***t***_**1**_ is the time of exposure, ***T***_**0**_ is the initial temperature of the sample equilibrated to the temperature of water in the tank.

Here, Equation (5b) takes into account the symmetry of the system and Equation (5c) assumes the thermostabilized border of the cuvette wall made of aluminum with high thermal conductivity. It is also assumed that the presence of NPs at the studied concentrations negligibly changes the specific heat capacity and density of water. At the US beam-suspension border, the boundary conditions are the temperature and heat flux equality:

(6a) $$\begin{array}{r} T_{1}{|}_{r\;=\;R_{\mathrm{beam}}}=T_{2}{|}_{r\;=\;R_{\mathrm{beam}}}; \end{array}$$

(6b) $$\begin{array}{r} -\kappa \;\frac{\partial T_{1}}{\partial r}{|}_{r=R_{\mathrm{beam}}}\;=-\kappa \;\frac{\partial T_{2}}{\partial r}{|}_{r=R_{\mathrm{beam}}}. \end{array}$$

In case of the suspension cooling succeeding the US-induced heating, the problem is reduced to the following set of equations:

(7a) $$\begin{array}{r} \frac{\partial T}{\partial t}\;=\;\chi \frac{\partial^{2}T}{\partial r^{2}},\;0<r<R_{\mathrm{cuv}}; \end{array}$$

(7b) $$\begin{array}{r} t=t_{1}:\;T=T\left(t_{1}\right),\;0\leq r\;\leq R_{\mathrm{cuv}}; \end{array}$$

(7c) $$\begin{array}{r} r=R_{\mathrm{cuv}}:\;T=T_{0},\;t_{1}<t\leq t_{2}, \end{array}$$

where ***T(t***_**1**_***)*** is the temperature distribution in the cuvette just after the heating phase, **(*t***_**2**_***-t***_**1**_**)** is the time of cooling.

The effective attenuation coefficients **α_*eff*_** of the polydisperse suspensions of PSi NPs were calculated for the distributions of particle sizes ***a***, which were measured using the DLS technique (see section Characterization of PSi NPs) and approximated using lognormal functions ***f(x)*** (Sviridov et al., 2013):

(8) $$\begin{array}{r} \alpha_{\mathrm{eff}}=\frac{\int f\left(a\right)\;\alpha \;\left(a\right)da}{\int f\left(a\right)da},\;f\left(x\right)=A+\frac{B}{\sqrt{2\pi }x\sigma }e^{-{\left(\ln x-m\right)}^{2}/2\sigma^{2}}, \end{array}$$

where the attenuation coefficient **α*(a)*** for each particle size can be calculated using Equation (2). The coefficients of lognormal functions for the size distributions of O-PSi NPs and J-PSi NPs are given at the bottom of Table S1.

Equation (4) was solved numerically using an implicit four-point difference scheme in MATLAB® (the Mathworks, Natick, MA) (Ames, 1992). The number of time and space mesh nodes was equal to 1,000 to provide an appropriate accuracy. An example of the radial temperature distribution calculated for the parameters specified in Table S1 is given in Figure S2 and is in accordance with the result one may predict: The heating effect dominates in the region of cuvette irradiated with the US and vanishes fast with the distance from the beam boundary.

Note, the temperature effect of US in the suspension of PSi NPs can be also analyzed by considering more sophisticated approaches, i.e., the Epstein, Carhart, Allegra, and Hawley (ECAH) theory, which takes into account the contributions from viscous and thermal transport processes at the interface of inhomogeneities, as well as intrinsic absorption in the components of the heterogeneous system (Allegra and Hawley, 1972). The dipole (first-order) contribution from the relative motion of spheres in respect to the molecules in the suspending fluid is predominant in case of large density differences between the suspended matter and that of the suspending fluid (even for materials with mean porosity of 40–60%, as it is valid for mesoporous silicon matrix). This first-order term is incorporated both into the ECAH model by including shear waves and into the model of Urick by using the Stokes calculation of the energy loss of a pendulum oscillating in a viscous fluid. At the other hand, the zero-order contribution, which takes into account the difference of attenuation between the substances comprising the suspension and heat flow between the particle and the suspending fluid, is 1–2 orders of magnitude smaller than the first-order contribution for rigid particles, by contrast with the case of emulsions, viscous suspending fluids and low-density particles like polystyrene (Allegra and Hawley, 1972). Therefore, we consider the results obtained by using the model of Urick (1948) quite satisfactory to analyze the US-induced heating dynamics in aqueous suspensions of PSi NPs.

## Results and Discussion

### Characterization of PSi NPs

**Figure 3:**
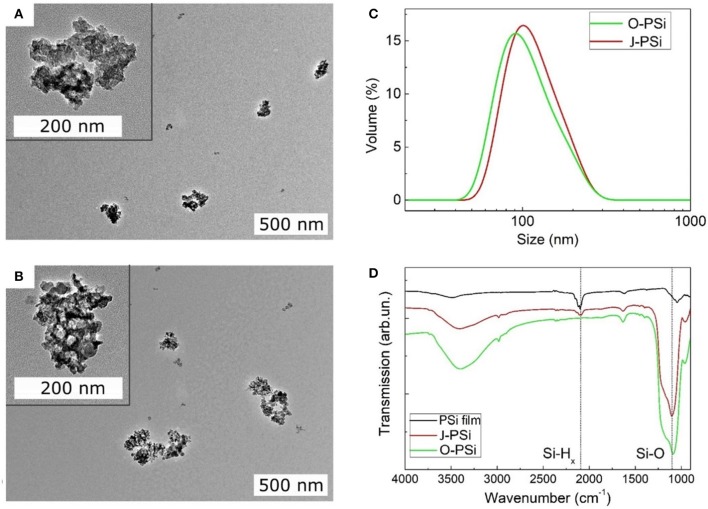
Material characterization of the PSi NPs. **(A,B)** The TEM images of TO-PSi NPs and J-PSi NPs, respectively; the insets show the NPs at a smaller scale. **(C)** Hydrodynamic size distributions of O-PSi NPs (green line) and J-PSi NPs (red line) in water. **(D)** FTIR spectra of the hydrogen terminated pristine PSi NPs (black line), O-PSi NPs (green line), and J-PSi NPs (red line).

Both the O-PSi and J-PSi NPs had irregular shapes (Figures 3A,B) due to the top-down fabrication method (Nissinen et al., 2016). The sizes of all the investigated NPs were in the range of 20–300 nm with the maximum close to 100 nm (Figure 3C), which are appropriate to provide temperature contrast with the surrounding medium under exposure to US, but are still small enough to reach distant tissues when considering their biomedical applications. FTIR measurements revealed that the pristine material of PSi displayed intense bands between 2,000 and 2,170 cm^−1^ due to the surface Si–H_*x*_ (*x* = 1–3) groups (Figure 3D) (Riikonen et al., 2012). After the complete surface oxidation (O-PSi), an intense peak at 1,000–1,200 cm^−1^ of Si–O_*x*_ bonds appeared, while the peak from Si–H_*x*_ disappeared completely. However, in the case of the J-PSi NPs, the intensity of Si–O_*x*_ was lower, and a small amount of Si–H_*x*_ bands was still present, indicating the success of production of J-PSi NPs to preserve Si–H groups on the inner pore surfaces.

### Measurement and Calculation of Temperature Evolution

The heating dynamics of the aqueous J-PSi NP suspensions was investigated under US radiation within 200 s, as well as the subsequent cooling dynamics for 300 s after the US radiation was turned off. The characteristic time of heat diffusion from a heated cylindrical volume of radius ***R***_***beam***_ = 5 mm can be estimated as:

(9) $$\begin{array}{r} \tau_{T}\;\approx \;\frac{R_{\mathrm{beam}}^{2}}{\chi }\;\approx \;175\;s. \end{array}$$

For the times considerably shorter than **τ_*T*_**, the temperature at the center of the cuvette grows approximately linearly after the US radiation is turned on. The process of heat diffusion becomes significant at the times exceeding **τ_*T*_**: The temperature increase slows down and its value reaches a constant level. As the US was turned off, the heated area spread over a distance comparable with ***R***_***beam***_, being less than ***R***_***cuv***_ = 12.5 mm. This estimation justifies the boundary conditions Equations (5c, 7c) of the heat-transfer equation, where the influence of metallic wall is negligible. To study in detail all the characteristic features of the US-induced heating and subsequent cooling of the suspensions, the US exposure time of 200 s and cooling observation time of 300 s were selected. The values of the temperature changes in the suspensions under the experimental conditions were about 1°C. To ensure a minimal measurement error, the initial temperatures of the suspension in the cuvette and the water in the tank were equalized with an accuracy of 0.1°C. Data from the thermocouple were recorded synchronously with the spectrum of the US wave transmitted through the cuvette at the frequency of 3 Hz.

**Figure 4:**
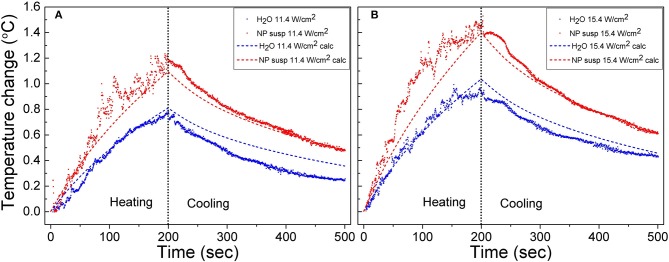
Raw heating and cooling curves for the suspension of J-PSi NPs and water obtained in the experiment under exposure to US with the intensities of 11.4 **(A)** and 15.4 W/cm^2^ **(B)** (shown by color points). Dashed curves correspond to the numerical calculation.

The raw experimental data and temperature curves calculated using Equation (4) for the suspensions of J-PSi NPs and distilled water under exposure to US with the intensities of 11.4 and 15.4 W/cm^2^ are given in Figure 4. The selected model describes the dynamics of heating with a sufficient degree of accuracy. At the US intensity of 11.4 W/cm^2^, the temperature rise in water and the suspension of J-PSi NPs is consistent with the theoretical calculation. However, there are small discrepancies close to the moment of US turn-off, when the maximum heating is observed. The presence of J-PSi NPs led to additional heating of the suspension by an average of 0.15°C. The temperature fluctuations in the suspension due to cavitation at a specified intensity of 11.4 W/cm^2^ were by 7–10% higher than fluctuations of the temperature in water. The decrease in temperature during the cooling phase occurred faster than it was predicted by the calculations both in the suspension and water (Figure 4A). Such behavior can be explained by the fact that the temperature in the tank was for some reason lower than the temperature of the suspension. As a result, there was a heat flow from the walls of the cuvette to its center, which resulted in faster cooling. The same effect led to a decrease in the peak temperature at the moment the US was switched off.

When the US intensity increases up to 15.4 W/cm^2^, the temperature fluctuations in the stage of heating grew both in water and in the suspension. The maximum range of temperature fluctuations was 0.4 and 0.3°C for the suspension and water, respectively, being about 30% of the peak temperatures. Cavitation also led to an increase in the average heating of both the suspension and water. However, the intensity of cavitation in the suspension of J-PSi NPs was higher, therefore, the additional heating in the suspension was, on the average, 0.2°C higher than in distilled water (Figure 4B). A satisfactory agreement between the measured and calculated values of temperature in the cooling stage at *t* > 300 s indicates that the temperature of the suspension in the cuvette and water in the tank were nearly equal at the initial time.

**Figure 5:**
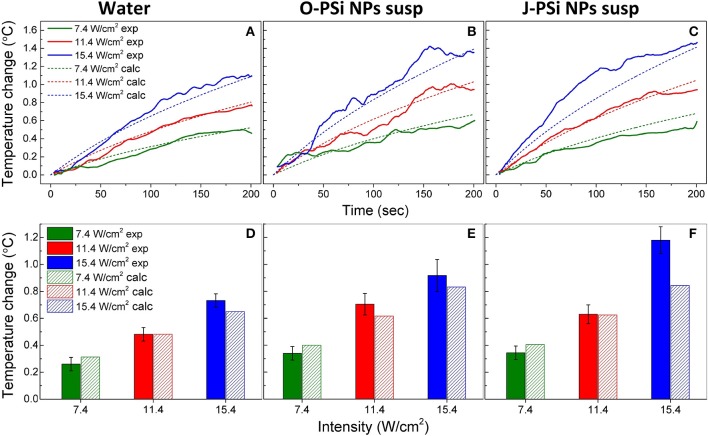
The temperature rise in distilled water **(A)**, the suspension of O-PSi NPs **(B)**, and the suspension of J-PSi NPs **(C)** at the concentration of 1 g/l induced by US with the intensities of 7.4, 11.4, and 15.4 W/cm^2^. Experimental results are shown by the solid lines, while the dashed lines represent the simulated data. Temperature increase in distilled water **(D)**, the suspension of O-PSi NPs **(E)**, the suspension and J-PSi NPs **(F)** after the first 100 s of exposure to US. The solid bars stand for the measured temperatures and the dashed bars for the calculated ones. The error bars indicate s.d. of the residual statistics obtained after non-linear fitting of the experimental heating curves (adj. *R*-squared > 0.98).

For a better interpretation of the obtained raw data, 30-point moving averages of the heating trends were plotted vs. time. Figures 5A–C shows the smoothed experimental and calculated time dependences of heating in distilled water (a), the suspensions of O-PSi NPs (b), and J-PSi NPs (c) at the concentration of 1 g/l induced by US with the intensities of 7.4, 11.4, and 15.4 W/cm^2^. The initial temperature of all the samples was ~23°C. The simulated curves for water are in a good agreement with the experimental data given in Figure 5A. The maximum heating of water at 15.4 W/cm^2^ amounted to 1.16°C and led to a negligible deviation from the calculated value of 1.09°C. Despite the smoothing, significant fluctuations of the experimental curves are still visible in Figure 5B. The most pronounced deviations from the results of simulation up to 0.1 and 0.2°C took place for the US intensities of 11.4 and 15.4 W/cm^2^, respectively. As expected based on Equation (2) (see section Cavitation Measurements), the maximum heating for the suspension of O-PSi NPs was higher than the one for the distilled water at both 11.4 W/cm^2^ (1.1°C) and 15.4 W/cm^2^ (1.4°C). The highest heating among all the samples at 15.4 W/cm^2^ was observed for the suspension of J-PSi NPs (Figure 5C) and amounted to 1.46°C. The maximum deviation from the simulated curve in 0.31°C was achieved 81 s after turning the US on. The fluctuations of the experimental curve at 11.4 W/cm^2^ were less pronounced and led to the difference of 0.1°C between the measured and calculated values.

For the further analysis, the temperature increase in water and the suspensions of O-PSi NPs and J-PSi NPs at the concentration of 1 g/l was plotted after 100 s of US exposure of different intensities (Figures 5D–F). The presented data unambiguously reveal contrast between the heating of distilled water and the suspensions of PSi NPs at the same US intensity, which could reach 0.4–0.6°C in the case of high intensity. Moreover, within the limits of error, the experimental values of heating at low intensities coincide with the simulated ones. However, at the highest intensity, the measured temperature increased significantly over the calculated values. In particular, for the suspension of J-PSi NPs the excess was 0.35°C, which can be explained by the prominent acoustic cavitation that led to the collapse of air bubbles accompanied by release of energy and additional heating (see section Cavitation Contribution to the Heating Effect).

**Figure 6:**
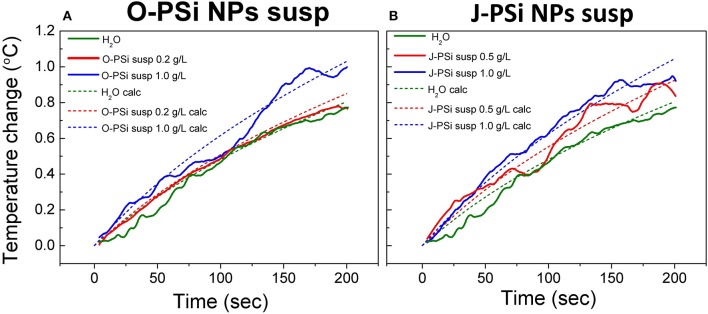
Experimental (solid lines) and theoretically calculated (dashed lines) time dependences of heating in the suspensions of O-PSi NPs **(A)** and J-PSi NPs **(B)** exposed to the US with the intensity of 11.4 W/cm^2^ at different NP concentrations.

Figure 6 represents the experimental and calculated time dependences of heating at different concentrations of nanoparticles in the suspensions of O-PSi NPs (a) and J-PSi NPs (b) at the US intensity of 11.4 W/cm^2^. At this intensity, the additional heating from cavitation was negligible (Figure 5E) and made it possible to investigate the effect of the PSi NPs concentration on the heating tendency. The concentrations of 0.5 and 1 g/l led to the temperature difference between the suspension of J-PSi NPs and distilled water in 0.15 and 0.3°C, respectively. The temperature difference between the suspension of O-PSi NPs at the concentration of 0.2 g/l and distilled water was almost negligible, while the concentration of 1 g/l led to the result equal to the case of J-PSi NPs. Such temperature increase was in good agreement with the increase of the US absorption coefficient described by Urick (1948). The calculated values of US absorption coefficients for distilled water, the suspensions of O-PSi NPs, and J-PSi NPs at 1 g/l were 0.108, 0.138, and 0.140 m^−1^, respectively. It should be noted, that these experiments were carried out with the concentration of PSi NPs in the suspensions up to 1 g/l, which defines the upper limit of concentration levels that are non-toxic to organisms (Näkki et al., 2015; Sviridov et al., 2017). High concentration levels of PSi NPs in tissues necessary for the contrast enhancement of ultrasound can be provided by the targeted delivery of nanoparticles. Only in this case it is possible to reach the desired effect using PSi NPs as the activators together with US of relatively low intensity (as compared, for example, with HIFU).

### Cavitation Contribution to the Heating Effect

**Figure 7:**
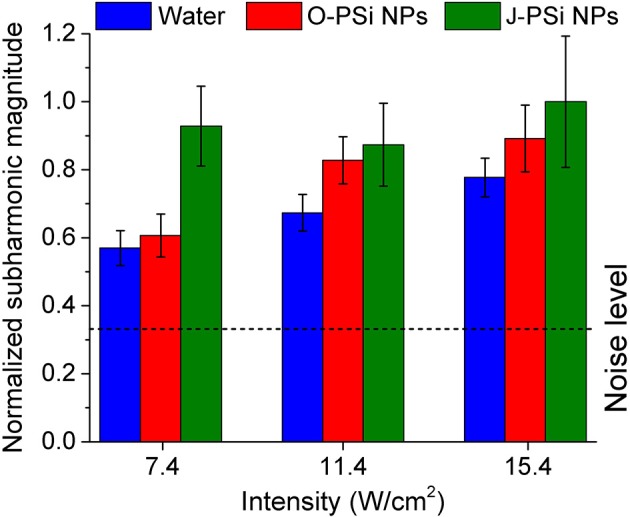
Mean subharmonic magnitudes in distilled water and the suspensions of O-PSi NPs and J-PSi NPs during 200 s of exposure to US at three different intensities. The dashed line corresponds to the noise level. The error bars indicate s.d. (significance level α = 5%).

Simultaneously with measuring the temperature during the heating process, the subharmonic magnitude in the spectrum of the transmitted signal was also measured, the generation of subharmonic characterizes the intensity of cavitation in the suspensions (Didenkulov et al., 2011). The values of mean subharmonic magnitudes for the distilled water and the NP suspensions during 200 s of exposure to US with the intensities of 7.4, 11.4, and 15.4 W/cm^2^ are shown in Figure 7. The subharmonic magnitudes for all the samples were much higher than the noise level, which means that the intensities mentioned were far beyond the cavitation thresholds (Tamarov et al., 2017). The subharmonic magnitude in water grew gradually with the US intensity: It increased by more than 30% when the intensity was changed from 7.4 to 15.4 W/cm^2^. The suspension of O-PSi NPs expressed also positive, but less progressive (as compared with water) trend in the subharmonic magnitude with the increase of US intensity. Furthermore, the magnitudes were higher as compared with such for the distilled water (0.89 against 0.78 μV at 15.4 W/cm^2^), which was associated with the greater number of the bubble nucleation centers when nanoparticles are present (Tuziuti et al., 2005; Sviridov et al., 2015). The subharmonic magnitude at the intensity of 15.4 W/cm^2^ was the highest one among for the J-PSi NPs. Here, the measured values of subharmonic magnitude in the suspension of J-PSi NPs at all the employed intensities coincided within the margin of error. This saturation can be explained with two reasons. The first factor is that at some limit value of US intensity the number of cavitating air bubbles stops increasing and so does the subharmonic magnitude. The second one is associated with the transmission geometry used (Figure 2). Even though the number of the cavitation bubbles collapsing per time unit increases with the US intensity (Tamarov et al., 2017) the growing number of the air bubbles leads to the scattering of the US signal detected by the hydrophone resulting in reduced subharmonic magnitude. It should be noticed, that besides the subharmonic generation the inertial cavitation above a threshold intensity should generate broadband white noise and harmonic emissions, which can be also responsible for additional heating effect.

**Figure 8:**
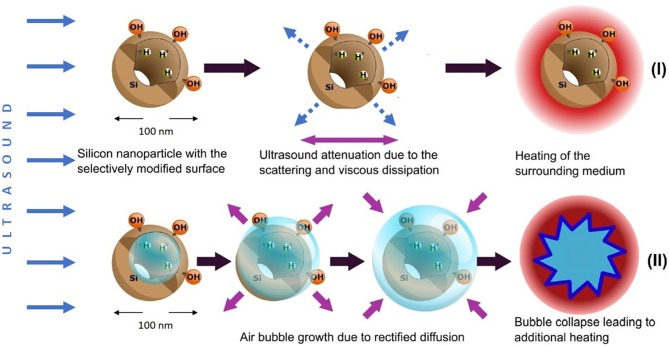
Two mechanisms of the dissipation of US energy in aqueous suspensions of O-PSi NPs and J-PSi NPs: (I) enhanced scattering of US waves and viscous dissipation of the US energy, which takes place for both types of the suspensions; (II) acoustic cavitation leading to the high-energy bubble collapse and additional heating, which is most pronounced in case of J-PSi NPs with hydrophobic inner walls. Blue solid arrows indicate energy flow vectors of the incident ultrasound beam, dashed arrows correspond to the waves scattered by PSi NPs. Violet arrows depict the process of viscous friction and air bubble dynamics.

Figure 8 gives a schematic view on two possible ways of the dissipation of US energy in the suspension of PSi NPs. For rather low US intensities (regime I) the main mechanism is determined by the US attenuation in the aqueous medium and the additional attenuation due to the solid particle-induced scattering and viscous dissipation (Sviridov et al., 2013). At higher US intensities, the acoustic cavitation leads to the high-energy bubble collapse and additional heating (regime II). The averaged thermal effect in regime I is well-estimated by using the model described in section Cavitation Measurements. Indeed, in this case the smoothed experimental heating curves are accurately approximated by the simulated ones (see green and red lines and bars in Figure 5). The discrepancies become observable for the heating curves of the NP suspension and distilled water (see red and blue lines in Figure 4, respectively). While the curve for water has week oscillations at 11.4 W/cm^2^, the presence of NPs in the aqueous medium leads to evident fluctuations. It became possible to observe such effects due to the usage of E-type thermocouple, which provided fast and precise control of temperature fluctuations with time resolution of ~200 ms evaluated using Equation (9). This value was close to the speed of data acquisition: the duration of a program cycle was ~300 ms.

The temperature fluctuations growing up to 0.1–0.3°C with the increase of the US intensity as well as the additional heating reaching the values of 0.3–0.4°C in the case of the high US intensities (see blue lines and bars in Figure 5), are attributed to the acoustic cavitation leading to vigorous bubble collapse and subsequent heat release. This phenomenon has a stochastic nature and threshold values, which depend on the number of cavitation nuclei (submicron air bubbles) (Li et al., 2017). PSi NPs are known to lower the thresholds of acoustic cavitation (Sviridov et al., 2015; Tamarov et al., 2017), which can be utilized to enhance the cell membrane permeability due to the effect of sonoporation (Mullin et al., 2013). The subharmonic magnitudes being the measure of cavitation activity (Figure 7) correlate well with the values of the heating: The heating trend exceeds the simulated curve because of the increase in the cavitation intensity. Moreover, the effect is stronger with J-PSi NPs, because the number of the cavitation nuclei in their suspension is the highest due to the residual air in hydrophobic pores when dispersed in water (Tamarov et al., 2017).

While the total thermal effect of PSi NPs under the employed NP concentration and US intensity is not enough to realize ultrasound hyperthermia itself, the cavitation related subsequences, which are induced by PSi NPs and especially the Janus-like ones, look promising for both the detection (by using the thermal and acoustic responses), and destruction of undesirable biological tissues and cells. The observed temperature fluctuations (see Figure 4) indicate the local heat release in the vicinity of the collapsing air bubbles growing from the pores of PSi NPs. The values of such cavitation-induced hyperthermia can exceed the average temperature of the surrounding medium (Zhou and Gao, 2013; Yoshizawa et al., 2017). This “nanoscalpel” effect (Osminkina et al., 2015) can be utilized for the targeted destruction of cancer cells, but in contrast to the HIFU technique, it employs clearly lower US intensities. Furthermore, outstanding opportunities for the surface modification of PSi NPs as US actuators open wide perspectives of auxiliary biomedical modalities like bioimaging (Kharin et al., 2015; Tolstik et al., 2016) and controlled drug release (Tamarov et al., 2016).

As for the ultrasound imaging, PSi NPs themselves are a weak contrast agent because they are too small to backscatter US waves at a detectable level. However, Janus-like PSi NPs look more promising for US imaging because they act as seeds for cavitation bubbles, which can be detected even with a standard US scanner (Tamarov et al., 2017).

## Conclusions

We investigated mesoporous silicon nanoparticles with fully oxidized and Janus-type hydrophobic/hydrophilic surfaces as potential contrast agents to stimulate the temperature increase of their aqueous suspensions. To achieve that, we developed an experimental setup to simultaneously measure the heating of the nanoparticle suspensions and monitor the cavitation intensity by measuring the subharmonic magnitude. Based on the measurements, we evaluated two main mechanisms leading to the heating effect in nanoparticle suspensions. The first mechanism was associated with enhanced scattering and viscous dissipation of the ultrasound energy in the aqueous medium filled with solid nanoparticles. This mechanism dominated for low ultrasound intensities and was numerically calculated using the model of heat transfer and ultrasound energy attenuation. The second mechanism was used to explain the discrepancies between the experimental and theoretical results at higher ultrasound intensities, as well as the differences between the suspensions of the oxidized and Janus-like nanoparticles. These discrepancies were because of the acoustic cavitation, which led to the high-energy bubble collapse. This contribution was observed by an apparent excess of the experimental heating curves over the simulated ones as well as strong temperature fluctuations. The increase of experimentally measured subharmonic magnitude in the spectrum of the acoustic signal transmitted through the cuvette correlated with the heating dynamics. The highest values of heating were obtained for the suspension of Janus nanoparticles, which inner pore walls were hydrophobic while the external surfaces were hydrophilic. Such surface modification made it possible to preserve nano-air seeds inside the pores of the nanoparticles, which acted as nuclei for the growth of the microbubbles and cavitation. The results of the present study obtained for the biocompatible and biodegradable porous silicon nanoparticles shed light on their behavior under ultrasonic radiation boosting comprehensively the development of inorganic nanoparticles for theranostic applications.

## Author Contributions

All authors listed have made a substantial, direct, and intellectual contribution to the work, and approved it for publication.

### Conflict of Interest Statement

The authors declare that the research was conducted in the absence of any commercial or financial relationships that could be construed as a potential conflict of interest.
