# Supplementary material for: Cavitation Induced by Janus-Like Mesoporous Silicon Nanoparticles Enhances Ultrasound Hyperthermia
Source: Front Chem. 2019 Jun 5;7:393. doi: 10.3389/fchem.2019.00393 (PMC6561312; doi:10.3389/fchem.2019.00393)
Supplement: Supplementary file 1 [file Data_Sheet_1.PDF]

Supplementary Material to paper entitled:

Cavitation Induced by Janus-like Mesoporous Silicon Nanoparticles Enhances Ultrasound Hyperthermia

Andrey Sviridov[1*], Konstantin Tamarov[1,2], Ivan Fesenko[1,3], Wujun Xu[2], Valery Andreev[1], Victor Timoshenko[1,3,4] and Vesa-Pekka Lehto[2]

[1]M.V. Lomonosov Moscow State University, Faculty of Physics, Moscow, Russia

[2]University of Eastern Finland, Department of Applied Physics, Kuopio, Finland

[3]National Research Nuclear University MEPhI, Institute of Engineering Physics for Biomedicine, Moscow, Russia

[4]Lebedev Physical Institute of the Russian Academy of Sciences, Moscow, Russia

*** Correspondence:**
Corresponding Author
asagittarius89@gmail.com

*** Correspondence:** Corresponding Author: asagittarius89@gmail.com

Numerical calculation of heating

Parameter	*Value*	*Description*
R_{cuv}	1.25 cm	cuvette radius
R_{beam}	0.5 cm	beam radius
N	1000	number of steps along the r-axis
t_1	200 s	time span for the heating process
$t_2 - t_1$	300 s	time span for the cooling process
T_0	23 °C	initial temperature

c_p	4200 J/kg·K	specific-heat capacity of water
c	1500 m/sec	speed of sound in water
κ	0.6 W/m·K	thermal conductivity of water
μ	0.01012 cm^2/sec	kinematic viscosity of water
ρ_0	1000 kg/m^3	density of water
ρ_1	2330 kg/m^3	density of silicon
$f = \omega/2\pi$	2.08 MHz	frequency of the sinusoidal US wave
α_1	$2.5\cdot10^{-2}$ m^{-1}	absorption coefficient of water for the frequency of 1 MHz
ν	0.1…1 g/l	concentration of nanoparticles in the suspension
I	5…20 W/cm^2	US intensity
A	-0.011<br>-0.024	TO-PSi NPs<br>SM-PSi NPs
B	1576.991<br>1738.878	TO-PSi NPs<br>SM-PSi NPs
σ	0.371<br>0.353	TO-PSi NPs<br>SM-PSi NPs

μ	4.738	TO-PSi NPs
	4.820	SM-PSi NPs

Table 1. Constants and parameters used in the numerical solution of heat transfer equation.

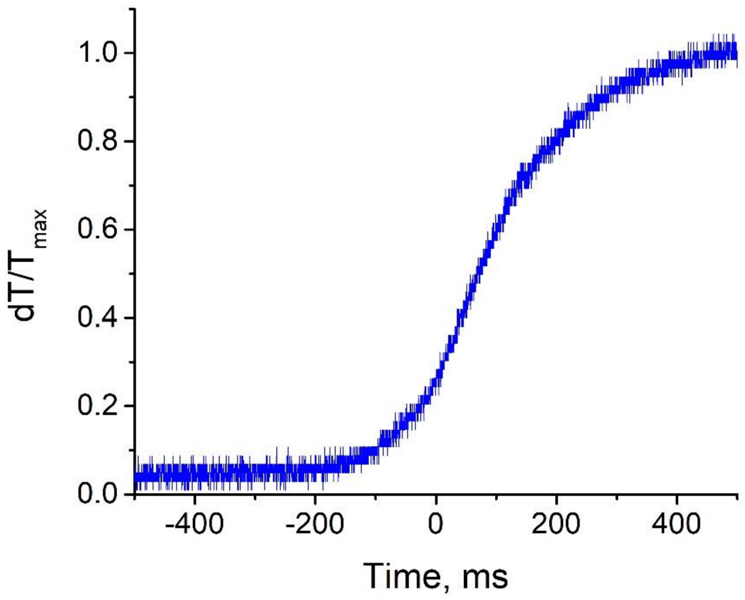

Figure S1. Transient characteristic of the thermocouple (TC). The magnitude of the time response was measured by rapidly placing the TC from air at a temperature of 23 °C into water heated to 30

°C. From the recorded dependence of temperature vs. time, the magnitude of the time response was determined at the point where the temperature was 0.63 of the maximum value.

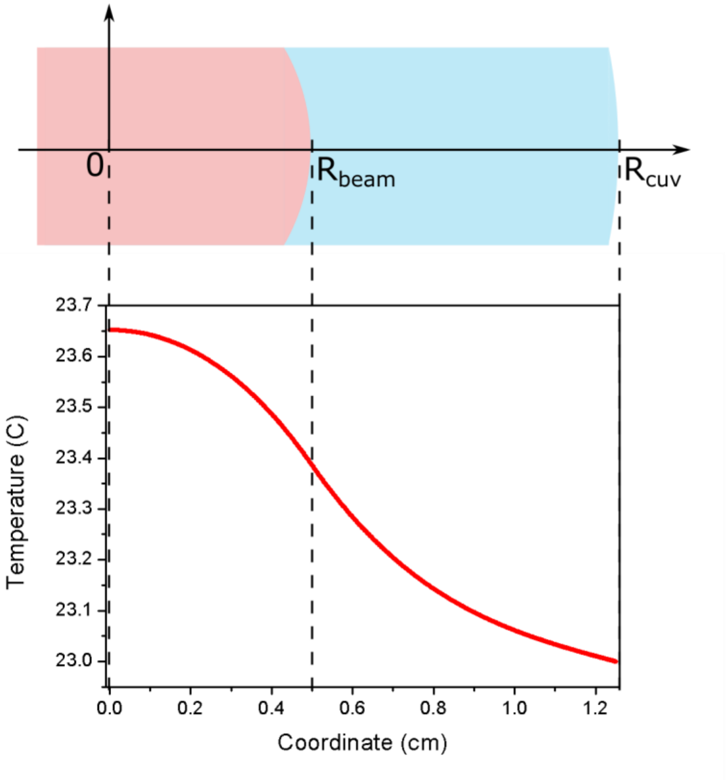

Figure S2. Geometrical model corresponding to the experimental setup which was used for the numerical solution of heat transfer equation (at the top). Example of the calculated radial temperature distribution in the suspension of PSi NPs at the concentration of 1 g/l (T_0 = 23 °C, I = 12 W/cm^2) after 100 s of US exposure (at the bottom).

Cavitation measurements

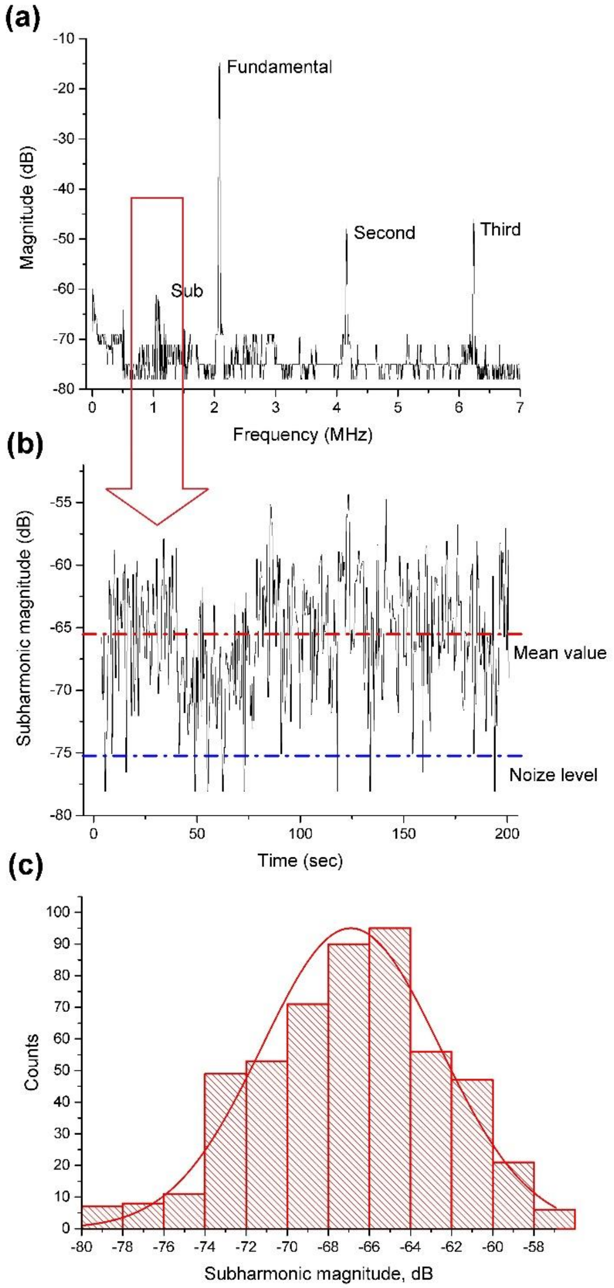

Figure S3. Technique of cavitation detection by measuring the subharmonic amplitude in the **(a)** signal spectrum. **(b)** The subharmonic amplitude (red dashed line) is higher than the noise level (blue dashed line). **(c)** The normal distribution of the subharmonic amplitude.

A custom-made piezoelectric transducer with diameter $d = 10$ mm was used in the measurements. The acoustic power W radiated by the transducer at the frequency of 2.08 MHz was determined by measuring the radiation force F_{rad} exerted on an absorbing target in water. The acoustic pressure P_a was calculated from the following relationship:

$$\mathbf{P}_{\boldsymbol{a}} = \frac{\boldsymbol{2c}}{\boldsymbol{d}}\sqrt{\frac{\mathbf{2}\boldsymbol{\rho F_{rad}}}{\boldsymbol{\pi}}},$$

where c = 1500 m/s is the speed of sound in water, ρ is the density. The pressure amplitude as a function of the peak-to-peak voltage applied to the transducer is shown in Figure S4.

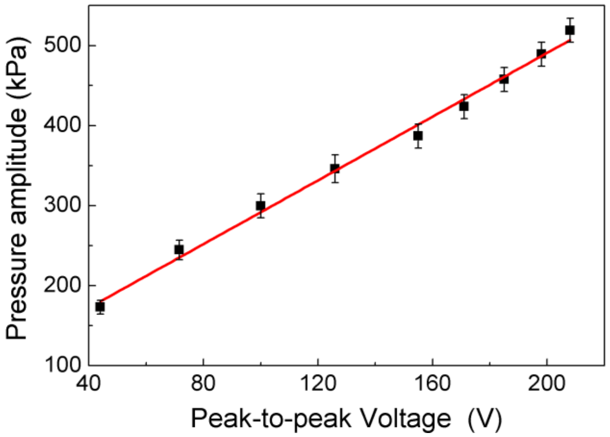

Figure S4. Dependence of the acoustic pressure on peak-to-peak voltage applied to the transducer. Symbols correspond to the measured data; the line is the linear approximation obtained by using a method of the least squares.
